# Expression of the immune checkpoint modulator OX40 indicates poor survival in acute myeloid leukemia

**DOI:** 10.1038/s41598-022-19972-1

**Published:** 2022-09-23

**Authors:** Maddalena Marconato, Joseph Kauer, Helmut R. Salih, Melanie Märklin, Jonas S. Heitmann

**Affiliations:** 1 Clinical Collaboration Unit Translational Immunology, German Cancer Consortium (DKTK), Department of Internal Medicine, University Hospital Tübingen, Tübingen, Germany; 2 DFG Cluster of Excellence 2180 ‘Image-Guided and Functional Instructed Tumor Therapy’ (IFIT), University of Tübingen, Tübingen, Germany; 3 Department of Immunology, Interfaculty Institute for Cell Biology, University of Tübingen, Tübingen, Germany; 4 Department of Oncology and Hematology, University Clinic Heidelberg, Heidelberg, Germany; 5 German Cancer Consortium (DKTK) and German Cancer Research Center (DKFZ), Partner Site Tübingen, Tübingen, Germany

**Keywords:** Haematological cancer, Acute myeloid leukaemia

## Abstract

Despite therapeutic advances, mortality of Acute Myeloid Leukemia (AML) is still high. Currently, the determination of prognosis which guides treatment decisions mainly relies on genetic markers. Besides molecular mechanisms, the ability of malignant cells to evade immune surveillance influences the disease outcome and, among others, the expression of checkpoints modulators contributes to this. In AML, functional expression of the checkpoint molecule OX40 was reported, but the prognostic relevance of OX40 and its ligand OX40L axis has so far not been investigated. Here we described expression and prognostic relevance of the checkpoint modulators OX40 and OX40L, analyzed on primary AML cells obtained from 92 therapy naïve patients. Substantial expression of OX40 and OX40L on AML blasts was detected in 29% and 32% of the investigated subjects, respectively, without correlation between the expression of the receptor and its ligand. Whereas OX40L expression was not associated with different survival, patients with high expression levels of the receptor (OX40^high^) on AML blasts survived significantly shorter than OX40^low^ patients (*p* = 0.009, HR 0.46, 95% CI 0.24–0.86), which identifies OX40 as novel prognostic marker and a potential therapeutic target in AML patients.

## Introduction

Acute Myeloid Leukemia (AML) is an aggressive neoplasia with high mortality rates^1^ . The prognosis of patients is highly heterogeneous^2,3^ and routinely assessed by analysis of genetic mutations and cytogenetic aberrations including, among others, mutations in NPM1, CEBPA, FLT3 and TP53 genes as well as cytogenetic abnormalities such as t(8;21), inv(16), inv(3), and del(5q)^4^. Based on their genetic profile, patients are categorized according to the National Comprehensive Cancer Network (NCCN) risk score into three different risk-groups^5^. However, despite an accurate genetic and cytogenetic classification, patients who are considered to have a favorable or intermediate risk often suffer from dismal disease course and early death. Identification of additional prognostic markers for AML, e.g. by flow cytometry, could support treatment choice and thus improve the quality of care for these patients^6,7^.

Many malignancies display abnormal expression of immune checkpoint molecules which enable evasion from tumor immune surveillance. So far, this has been extensively investigated for the inhibitory checkpoints PD-1, CTLA-4, TIM-3 and LAG-3 on solid tumors^8^, but the same pathophysiological principle holds true for hematologic malignancies, and we recently reported on the functional role of the immune checkpoint modulator OX40 in AML^9^.

OX40 is a member of the TNF receptor family, which is mainly known to promote effector T cell differentiation, proliferation, long-term survival, and pro-inflammatory cytokines production, while inhibiting differentiation and suppressive activity of regulatory T cells (Tregs)^10–12^. We recently showed that signaling via its ligand OX40L expressed by NK cells promotes their activation, cytokine production and cytotoxicity^9^. Expression of OX40 on tumor cells and tumor infiltrating lymphocytes has been investigated in solid cancers, with at least partially conflicting results regarding its prognostic relevance^13–15^. Despite our previous findings about the expression of OX40 in AML, it is still unknown if OX40L is also expressed. Furthermore, the prognostic relevance of these markers has not been described.

Here we report on the expression of OX40 as well as its ligand OX40L in AML providing data obtained by analyzing molecular characteristics as well as disease outcome in 92 AML patients.

## Results

### Clinical characteristics of the AML patient cohort

OX40 and OX40L expression were analyzed using primary AML samples of 92 patients. The clinical characteristics of the patients are given in Table 1. Of all analyzed patients, 31 presented with undifferentiated leukemia (M0: n = 9, M1: n = 22), 22 with immature granulocytic leukemia (M2: n = 16, M3: n = 6), and 38 with monocytic leukemia (M4: n = 17, M5: n = 21); one patient could not be classified according to the FAB classification. In 70 and 22 patients, primary AML and secondary AML was diagnosed, respectively. Patient age ranged from 21 to 86 years (with a median of 59 years) with a female: male ratio of 1:1.36. Patients comprised 6 cases with t (15; 17) and 4 with inv(16). FLT3-ITD and FLT3-TKD mutations were detected in 28 and 5 patients, respectively. NPM1 mutations were observed in 24 patients, while CCAAT/enhancer binding protein α (CEBPA) mutations, MLL-AF9 fusion gene and IDH2 mutation were seen in 6, 2 and 6 patients, respectively. Based on cytogenetics, patients were categorized according to National Comprehensive Cancer Network (NCCN) risk score. Patient numbers in the favorable, intermediate and poor risk group were 33, 36 and 16, respectively. Seven patients could not be classified.

**Table 1:** Patient characteristics.

	Number of patients (%)
**Sex**	
Male	53 (58)
Female	39 (42)
Median age (years)	59 (range 21–86)
**FAB classification**	
M0	9 (10)
M1	22 (24)
M2	16 (17)
M3	6 (7)
M4	17 (18)
M5	21 (23)
Not classified	1 (1)
**WHO classification**	
AML with recurrent genetic abnormalities	55 (60)
AML with myelodysplasia-related changes	11 (12)
Therapy-related myeloid neoplasms	3 (2)
Myeloid neoplasms with germline predisposition	0 (0)
AML. not otherwise specified	24 (26)
**Primary/secondary AML**	
Primary	70 (76)
Secondary	22 (24)
**Blood count (median)**	
WBC (G/L)	56.9 (range 4.6–448.3)
Hb (g/dl)	8.6 (range 4.4–12.9)
Plt (G/L)	43.5 (range 6–252)
**NCCN risk score distribution**	
Favorable	33 (36)
Intermediate	36 (39)
Poor	16 (17)
Not classified	7 (8)
**Genetic abberations**	
t(15;17)	6 (6)
inv(16)	4 (4)
FLT3-ITD	28 (26)
FLT3-TKD	5 (5)
NPM1	24 (21)
CEBPA	6 (6)
MLL-AF9	2 (2)
IDH 2	6 (6)

*AML* acute myeloid leukemia; *FAB* French-American-British; *WBC* white blood count; *Hb* hemoglobin; *Plt* thrombocytes; *NCCN* National Comprehensive Cancer Network.

### OX40 and OX40L expression on primary AML cells

**Figure 1:**
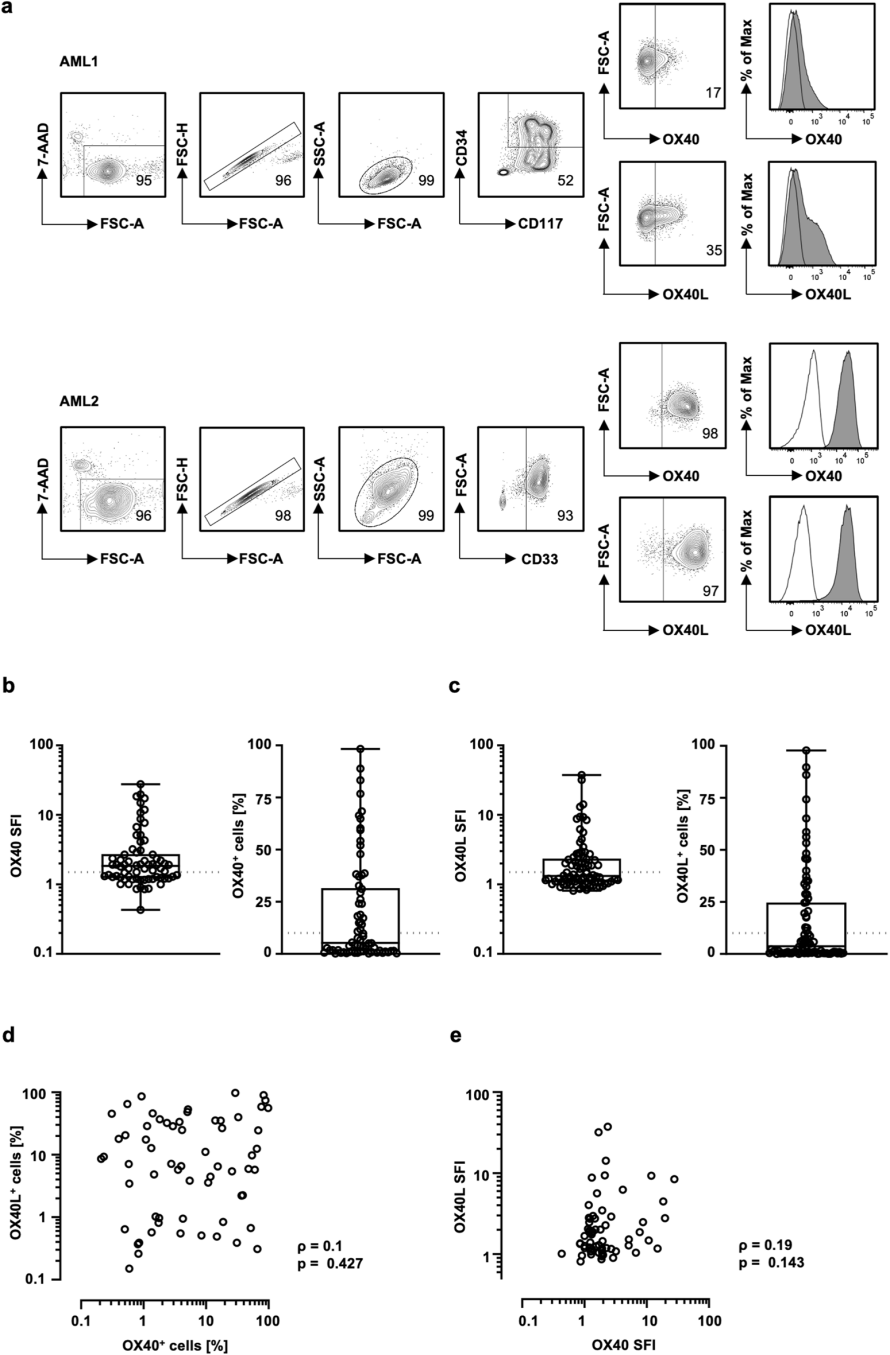
OX40 and OX40L expression on hematopoietic cells. OX40 expression was analyzed on hematopoietic cells by flow cytometry. (**a**) Gating strategy for two exemplary AML samples is outlined: viable (7-AAD^−^), singlets, mononuclear cells, blast marker (AML1: CD34^+^/CD117^+^. AML2: CD33^+^) and OX40 or OX40L expression as percentage. The histogram shows the representative OX40/OX40L staining (filled peaks) and the corresponding isotype control (open peaks). (**b**) OX40 expression on blasts of AML patients are depicted as SFI levels (analyzed patients n = 70, positive patients n = 41) and percentage of OX40 positive blasts (analyzed patients n = 70, positive patients n = 30) (boxplots with min/max whiskers). SFI levels and percentage of positive cells above 1.5 and 10%, respectively, were considered as positive expression (dotted line). (**c**) OX40L expression on blasts of AML patients are depicted as SFI levels (analyzed patients n = 86, positive patients n = 37) and percentage of OX40 positive blasts (analyzed patients n = 86, positive patients n = 30) (boxplots with min/ max whiskers). (**d, e**) Correlation between the expression of OX40^+^ and OX40L^+^ cells as percentage (**d**) and SFI (**e**) (*p* value and spearmans ρ).

Leukemic blasts were analyzed for OX40 and OX40L expression by flow cytometry as exemplified in Fig. 1a. Defining an SFI level of 1.5 as a margin for positivity, 29% and 32% of AML patients were found to express OX40 and OX40L, respectively (Fig. 1b,c). When the frequencies of OX40 positive and of OX40L positive cells (> 10%) were considered, 21% of AML patients were found to express OX40 and 21% OX40L (Fig. 1b,c). Among patients, highly variable surface levels of OX40 and OX40L were observed, reaching SFI levels up to 27.25 and up to 36.59, respectively. In addition, the percentage of OX40 and OX40L positive cells varied substantially for both molecules, ranging from 0% to almost 100% (Fig. 1b,c). No correlation between the expression of OX40 and OX40L on AML blasts was observed, both in terms of SFI and percentage of positive cells (Fig. 1d,e).

### OX40 and OX40L expression and patients’ clinical and genetic features

**Figure 2:**
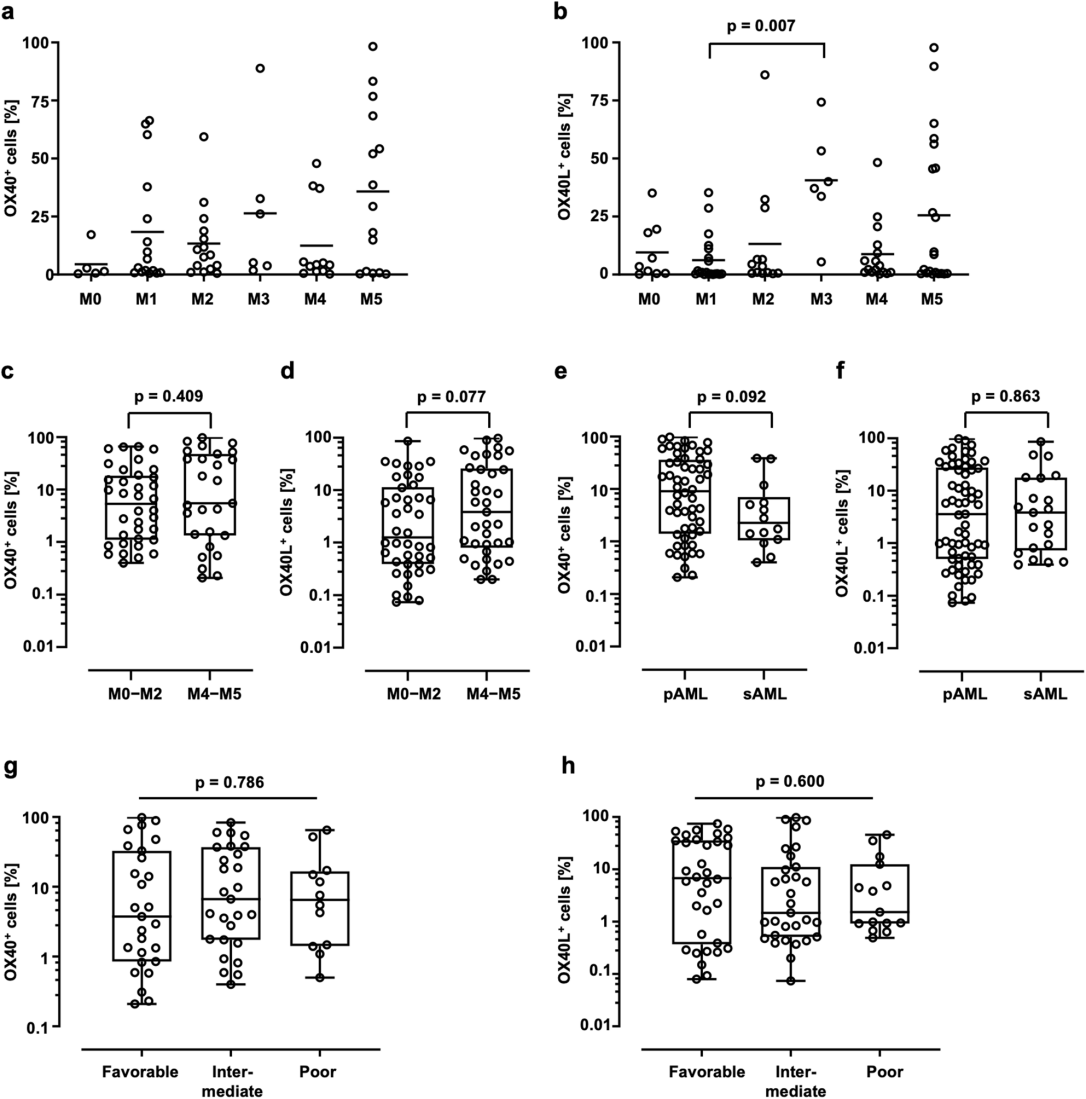
OX40 and OX40L expression on primary AML cells and association with clinical parameters. Frequencies of OX40 (**a**) and OX40L (**b**) positive blasts according to the different FAB classifications (single values, median, Kruskal–Wallis test) are showed. The expression of OX40 (**c**) and OX40L (**d**) was analyzed on FAB M0-M2 vs. FAB M4-M5 (min/max whiskers, Mann–Whitney-U test). The expression of OX40 (**e**) and OX40L (**f**) was analyzed primary (pAML) vs. secondary (sAML) AML (min/max whiskers, Mann–Whitney-U test). Distribution of OX40 (**g**) and OX40L (**h**) expression (%) throughout NCCN risk group (min/max whiskers, Kruskal–Wallis test) is showed.

Frequencies of OX40 positive cells differed among FAB subclasses (Fig. 2a), with a tendency to higher expression in subjects presenting with mature leukemia (FAB M5). Similarly, the frequency of OX40L positive cells varied among FAB subclasses, with a tendency to higher expression in subjects presenting with acute promyelocytic leukemia (FAB M3) and mature leukemia (FAB M5) and a significantly higher expression in FAB M3 compared to FAB M1 (Fig. 2b). No differences in OX40 and OX40L expression were observed when AML patients were grouped according to their myeloid (M0-M2) or monocytic (M4, M5) signature (Fig. 2c,d). Expression of OX40 and OX40L did not differ significantly between primary (pAML) and secondary AML (sAML) cases (Fig. 2e,f). When grouped according to NCCN risk classification, no difference in terms of OX40 and OX40L expression was observed among patients with intermediate, favorable, or poor risk (Fig. 2g,h).

The expression of OX40 and OX40L in patients with different genetic background was investigated (Table 2). Patients with FLT3-ITD mutation presented with higher OX40 expression levels (both SFI and percentage of positive cells) than FLT3 wild-type subjects. Moreover, OX40L expression levels (SFI and percentage of positive cells) were significantly higher in patients carrying translocation t(15:17) than in patients who did not present it, whereas CEBPA-mutated patients had lower expression levels of OX40L on leukemic blasts than CEBPA-wild type ones.

**Table 2:** Genetic background of AML patients and expression of OX40, OX40L.

Marker	Characteristics	N	Mutated/positive (mean + /− SD)	N	Unmutated/negative (mean + /− SD)	*p* value
OX40 SFI	t(15;17)	6	3.4 (4.1)	63	3.6 (5.2)	0.639
	inv(16)	4	2.2 (1.9)	63	3.7 (5.3)	0.525
	**FLT3-ITD**	**28**	**5.0 (5.8)**	**36**	**2.8 (4.7)**	**0.025**
	FLT3-TKD	5	1.7 (0.4)	59	3.9 (5.4)	0.950
	NPM1	24	5.8 (7.6)	40	2.4 (2.5)	0.131
	CEBPA	6	1.7 (0.6)	51	3.5 (4.7)	0.876
	MLL-AF9	2	1.0 (0.2)	59	3.4 (4.4)	0.096
	IDH 2	6	2.8 (2.9)	25	2.2 (2.4)	0.484
OX40%	t(15;17)	6	26.3 (33.2)	63	18.7 (25.1)	0.297
	inv(16)	4	13.6 (22.9)	63	20.3 (26.2)	0.443
	**FLT3-ITD**	**28**	**28.7 (28.2)**	**36**	**13.1 (23.4)**	**0.020**
	FLT3-TKD	5	5.4 (7.5)	59	26.6 (27.1)	0.220
	NPM1	24	27.6 (30.2)	40	14.5 (22.9)	0.149
	CEBPA	6	7.1 (9.0)	51	19.3 (26.4)	0.507
	MLL-AF9	2	2.1 (1.9)	59	19.0 (25.2)	0.331
	IDH 2	6	17.9 (22.1)	25	14.2 (25.0)	0.881
OX40L SFI	**t(15;17)**	**6**	**3.5 (2.8)**	**63**	**3.0 (5.7)**	**0.025**
	inv(16)	4	2.2 (1.1)	63	3.1 (5.8)	0.220
	FLT3-ITD	28	3.3 (6.6)	36	2.9 (5.0)	0.516
	FLT3-TKD	5	3.7 (5.1)	59	3.1 (5.8)	0.770
	NPM1	24	3.0 (6.4)	40	3.2 (5.2)	0.224
	CEBPA	6	1.3 (0.6)	51	3.5 (6.3)	0.061
	MLL-AF9	2	1.1 (0.2)	59	3.2 (5.9)	0.268
	IDH 2	6	6.2 (12.3)	25	4.1 (7.4)	0.530
OX40L %	**t(15;17)**	**6**	**40.6 (22.7)**	**63**	**13.5 (2.2)**	**0.004**
	inv(16)	4	19.0 (16.6)	63	14.8 (23.5)	0.126
	FLT3-ITD	28	15.1 (23.5)	36	15.5 (23.6)	0.977
	FLT3-TKD	5	21.6 (26.3)	59	14.9 (23.3)	0.956
	NPM1	24	14.2 (22.8)	40	16.1 (24.0)	0.588
	**CEBPA**	**6**	**5.1 (10.2)**	**51**	**17.5 (24.7)**	**0.007**
	MLL-AF9	2	2.19 (3.09)	59	15.4 (23.4)	0.245
	IDH 2	6	22.6 (33.5)	25	23.9 (29.5)	0.962

Significant values are in bold.

### Association between OX40 and OX40L expression and clinical outcome

**Figure 3:**
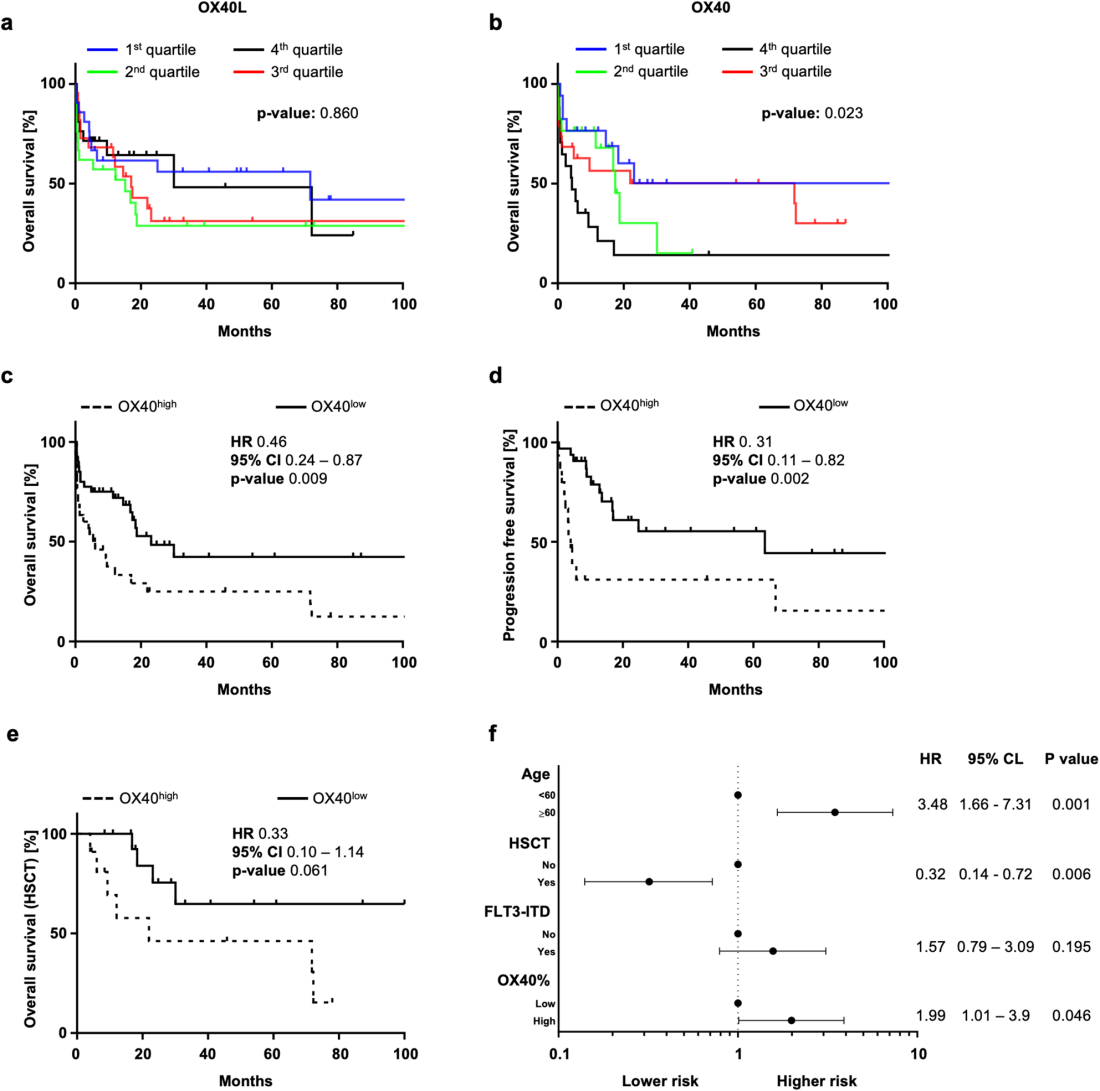
Impact of OX40 and OX40L expression on clinical outcome. The Overall Survival (OS) in AML patients is shown according to OX40L (**a**) and OX40 (**b**) expression (4 quartiles identified between minimal and maximal expression) in Kaplan–Meier analysis. OS in patients with OX40^high^ and OX40^low^ expression is shown (Kaplan–Meier analysis) (**c**). Median OS was reached in OX40^high^ (dotted line) and OX40^low^ (continuous line) patients after 6 and 23.2 months, respectively, and differed significantly (log-rank test). Progression-free survival (PFS) is shown according to OX40 expression in Kaplan–Meier analysis (**d**). PFS of OX40^high^ (dotted line) and OX40^low^ (continuous line) patients was 4.2 and 63.5 months, respectively, and differed significantly (log-rank test). OS of OX40^high^ (dotted line) and OX40^low^ (continuous line) transplanted patients is shown (Kaplan–Meier analysis) (**e**). Median OS was reached for OX40^high^ patients after 21.97 months. A multivariate survival analysis (cox regression) including the expression of OX40 (high. vs low), patients’ age (< 60 vs. > = 60 years), FLT3-ITD and the ASCT status of 65 AML patients was performed (**f**). HR = Hazard ratio, 95% CI = 95% confidence interval.

Next, we analyzed the association of OX40 and OX40L expression on AML cells with patient overall survival (OS) by a stratification of patients into four quartiles, according to the frequency of OX40 and OX40L positive cells. No OS difference was observed for patients displaying differing levels of OX40L expression (Fig. 3a). On the contrary, the OS differed significantly among the patients grouped into the different OX40 quartiles (*p*-value 0.023, with subjects in the fourth quartile (highest expression of OX40) presenting with the shortest OS (Fig. 3b). As the expression of OX40 associated with a negative outcome, the survival of all patients was further analyzed by using a calculated cut-off to group patients into OX40^high^ and OX40^low^ cases. Patients in the OX40^high^ group were found to have survived significantly shorter compared to the OX40^low^ group (*p*-value 0.009, HR 0.46, 95% CI 0.24–0.87) (Fig. 3c). The worse outcome of OX40^high^ patients was further confirmed in terms of progressive free survival (PFS), with OX40^high^ subjects presenting a significantly shorter PFS compared to the OX40^low^ cases (*p*-value 0.002, HR 0.31, 95% CI 0.11–0.82) (Fig. 3d).

Therapy regimes, i.e. anthracycline-based induction therapy, allogenic transplantation (ASCT) as well as supportive regimes, were similarly distributed in OX40high and OX40 low cases (Sup Table  1). Furthermore, when patients were grouped according to OX40 expression in quartiles, no differences were observed (sup. Table 2). Of note, OX40^high^ subjects after ASCT showed a trend towards a shorter OS comparted to OX40^low^ subjects (*p*-value 0.061, HR 0.33, 95% CI 0.10–1.14) (Fig. 3e). In addition, in a multivariate survival analysis including OX40 expression (high vs low expression), patients’ age (< 60 vs. ≥ 60 years), FLT3-ITD mutational status and ASCT status, OX40 expression on AML blasts was identified as independent risk factor for dismal outcome (Fig. 3f).

## Discussion

The TNFR family member OX40 is an activating receptor that sustains T cell anti-tumor reactivity upon binding to OX40L, thereby acting as a stimulatory immune checkpoint. The prognostic relevance of the expression of OX40 and its ligand has been investigated in solid tumors. Results were at least partially conflicting in different neoplasia, as OX40 expression is associated with better survival and response to treatment, e.g. in ovarian cancer^13^, but also with more aggressive diseases, e.g. in breast cancer^14,15^. In AML, the impact of OX40 and OX40L expression has so far not been investigated in detail.

Here we analyzed the expression of OX40 and OX40L on peripheral blood blasts of 92 AML patients. Our results confirmed that AML cells express OX40 in a substantial proportion of cases and demonstrate that also its ligand, OX40L, can be expressed by AML cells. In our cohort, frequencies of OX40 and OX40L positive AML cases were 29% and 32%, respectively. Of note, no correlation between expression of OX40 and its OX40L on AML blasts was observed.

To gain further insight into the clinical relevance of OX40 and OX40L, patients were grouped according to various laboratory and clinical markers. Besides a trend towards a higher expression of OX40 and OX40L in patients with FAB M3 and M5 AML cases, neither molecule was differentially expressed in any of the FAB subtypes, and a significant difference of OX40L expression could be observed only in M3 patients compared to M1. In terms of patients’ age and disease origin (primary vs secondary AML), a higher expression of OX40 was identified in primary AML only with regards to SFI (Figure S1), while the frequencies of positive cells were found to be homogeneous for both markers in each group of patients. In addition, OX40 and OX40L positive patients were similarly distributed among the NCCN cytogenetic risk-groups. Nevertheless, an association between OX40 expression and the high-risk mutation FLT3-ITD was observed, which is consistent with recent results of a screening of the Cancer Genome Atlas (TCGA) transcriptome data conducted by Gu et al.^16^. Furthermore, patients presenting with the favorable risk mutation CEBPA had a lower expression of OX40L compared to CEBPA negative patients. Patients carrying the translocation t (15;17) exhibited higher levels of OX40L compared to t(15;17) negative patients, which is in line with the higher expression seen in M3 cases as described above. To our knowledge, this is the first time that such results are reported, and a validation by further analysis of larger cohorts of patients and by including other molecular approaches is certainly warranted.

Next, we analyzed whether the survival of patients associated with the expression of OX40 or OX40L and found no association between OX40L and disease outcome. On the other hand, the expression of the OX40 checkpoint molecule on AML blasts strongly associated with patients’ outcome and OX40^high^ patients were found to have a significantly shorter survival compared to OX40^low^ subjects, which held true both in terms of OS and PFS, thus representing the first description of the prognostic relevance of OX40 in AML. This association between the expression of OX40 at the molecular level and unfavorable disease outcome appears to corroborate the observation of Gu et al.^16^, who likewise reported a poorer outcome for AML patients with documented RNA expression of the *OX40* gene in a retrospective TCGA analysis^16^. Furthermore, a multivariate survival analysis confirmed that OX40 represents a risk factor for shorter OS independently of age, FLT3-ITD and allogenic transplantation.

The mechanisms leading to the poorer outcome of OX40 positive AML patients so far remain unclear and warrant the conduct of detailed functional studies. The observation is, however, in accordance with our previously reported findings that signals transduced by OX40 into AML cells promote cells proliferation and survival, potentially due to an observed induction of cytokines that favor survival of leukemic cells^9^. Consistently, the interaction with either soluble or cell-bound OX40L might trigger the OX40 signaling, with OX40L being possibly provided by various cells of the immune system, but also by leukemic cells, even if no correlation between OX40 and OX40L expression on the very same AML blasts of a given patient was observed in our analysis. With this regard, an auto-stimulatory loop can be postulated at least for OX40 and OX40L double positive cells. However, further data are required to confirmed this hypothesis.

Other investigators reported that the expression of OX40 is associated with a mutated state of TP53, both in breast cancer^17^ and AML^16^, thus suggesting that OX40 is a hallmark of genetically more aggressive diseases. However, the reported association of high expression of the OX40 gene with shorter OS held true also for AML patients with wild type TP53 and FLT3^16^. Together with our results, this indicates that OX40 expression correlates with a more aggressive disease regardless of the genetic background, possibly by effects on cell proliferation and survival and potentially also yet unidentified immune-mediated mechanisms. It is for example tempting to speculate that AML cells expressing OX40 might compete with T cells for the binding to OX40L, thus preventing the immune system to mount a sustained anti-tumor response.

Overall, our findings not only identify OX40 as novel prognostic parameter that might serve to better guide treatment decisions in AML. They are also of high relevance in light of approaches aiming to develop therapeutic options to modulate the OX40/OX40L molecular system and which could be implemented also in the treatment of AML patients. The so far conducted pre-clinical studies have evaluated agonistic OX40-antibodies, which were found to promote tumor regression in melanoma-, colon carcinoma-, and glioma-models, by reinforcing the immune response against the malignant cells^18–20^. Consistently, promising clinical and immunologic results in terms of T-cell activation were achieved in two Phase I trials investigating 9B12 and MEDI6469, OX40-agonist antibodies, in patients with metastatic solid cancers and locally advanced neck squamous cell carcinoma^21,22^. Furthermore, several OX40 agonistic antibodies are being evaluated in early clinical trials alone or in combination with other checkpoints inhibitors for the treatment of solid cancers^23,24^. On the contrary, results of only one phase I trial in relapsed/refractory AML patients investigating an OX40 agonist are available, showing a favorable safety profile, but failed to achieve any response^25,26^. This is of particular interest in light of the findings presented in the present study and our previous results on the functional role of OX40 signaling into AML cells^9^, which shed light into a potential dual role of the OX40/OX40L axis that needs to be considered when designing clinical studies, e.g. by using targeted approaches like bispecific antibodies allowing to restrict agonistic effects to the desired immune stimulation, while preventing undesired survival modulation by OX40 signaling into AML cells that could promote the disease and impair patients’ survival.

In conclusion, to our knowledge, this is the first study reporting on the clinical relevance of the expression of both OX40 and OX40L in AML patients. The observed significant association of OX40 with shorter OS and PFS highlights the suitability of OX40 as prognostic marker for AML and provides important information for approaches aiming to engraft OX40 modulation for immunotherapy of cancer.

## Methods

### Patients’ samples and clinical characterization

Peripheral blood samples of 92 patients with AML were obtained at primary diagnosis. Peripheral blood mononuclear cells (PBMC) of patients were isolated by density gradient centrifugation and used for flow cytometry. Median observational time for all patients was 12.16 months (IQR 1.52–32.96 months). Diagnosis and classification of AML samples relied on morphology and cytochemistry of bone marrow according to the French-American-British (FAB) classification^27^. Cytogenetic and molecular analyses were performed at the University of Ulm with standard methods. All analyses were performed in agreement with the local ethic committee (project number: 13/2007 V) as well as with relevant regulation and guidelines of the University of Tuebingen. Patients signed an informed consent prior to study inclusion and all the analysis have been performed in accordance with the Declaration of Helsinki.

### Flow cytometry

PBMC of AML patients (0.5 × 10^6^ cells per staining) were incubated in medium containing human IgG (10 μg/ml, Sigma-Aldrich, St. Louis, MO) to prevent unspecific Fc-receptor binding prior to the staining, then washed and incubated with unconjugated OX40 and OX40L mAb (clone BerAct35 and ANC10G1, Ancell, Stillwater, MN) or isotype control at 10 μg/ml, followed by species-specific PE-conjugated antibodies (1:100, Jackson ImmunoResearch, West Grove, PA). After staining for expression for OX40/OX40L, AML blasts within PBMC were identified according to the immune phenotype obtained at diagnosis by simultaneous staining for CD33-PacificBlue, CD34-APC, CD38-FITC, CD117-PE-Cy7, and CD13-APC-Cy7 with an antibody staining cocktail. Fluorescence-conjugates (CD13, CD33, CD34, CD38, and CD117, BioLegend, San Diego, CA) were used in 1:100–1:200 dilutions. Dead cells were excluded based on 7-AAD (1:200, BioLegend, San Diego, CA) positivity. Specific fluorescence indices (SFIs) were calculated by dividing median fluorescence obtained with anti-OX40 and OX40L mAbs by median fluorescence obtained with the IgG_1_ isotype control. Positive expression was defined as SFI ≥ 1.5. Measurements were conducted using an LSR Fortessa or a FACSCanto II (BD Biosciences, Heidelberg, Germany), and for every sample at least 100,000 cells were recorded and used for further gating as shown in Fig. 1A. Data analysis was performed with FlowJo_V10 software (FlowJo LCC, Ashland, OR).

### Statistical analysis

Data are shown as mean ± SD and boxplots including median and 25% and 75% quartiles as well as min/max whiskers. To compare individual groups, the 2-tailed unpaired Mann–Whitney test or Kruskal–Wallis test were used. Patients with missing genetic data were excluded in the respective analysis. The correlation between the expression of OX40 and OX40L was analyzed using spearman’s rank correlation coefficient. Distribution of overall survival (OS) was calculated using the Kaplan–Meier method. Log-rank test was performed to compare survival between groups. For predictive cut-off value estimation, we sub-grouped OX40 and OX40L with respect to corresponding OS times using receiver-operating characteristics (ROC) analysis. Value of highest Youden index was used as cut-off. Cut-off values of 10.79% enabled further separation of cases in OX40^high^/OX40^low^ according to the frequency of OX40 positive cells. Statistical analyses were conducted using JMP® Pro (SAS Institute, Version 14.2) and GraphPad Prism 9.1.2 software. P values of < 0.05 were considered statistically significant.

## Supplementary Information

Supplementary Information.

## Data Availability

The corresponding author had full access to all the data in the study and all authors shared final responsibility for the decision to submit for publication. The datasets generated during the current study are available from the corresponding author on reasonable request.
