# Supplementary material for: Expression of the immune checkpoint modulator OX40 indicates poor survival in acute myeloid leukemia
Source: Sci Rep. 2022 Sep 23;12:15856. doi: 10.1038/s41598-022-19972-1 (PMC9508266; doi:10.1038/s41598-022-19972-1)
Supplement: Supplementary file 1 — Supplementary Information. [file 41598_2022_19972_MOESM1_ESM.docx]

**
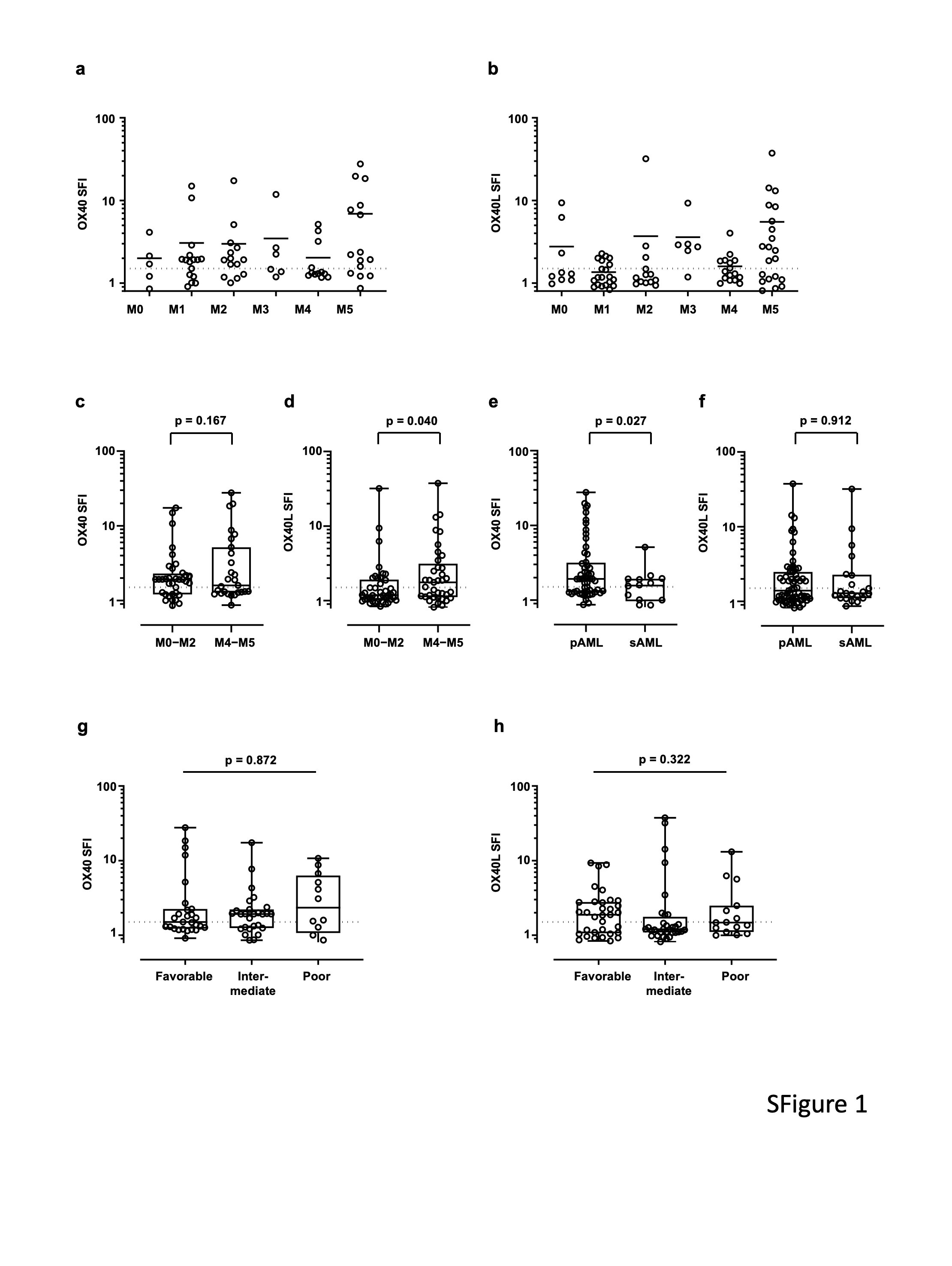
**

**Figure S1.** OX40 and OX40L expression on primary AML cells and association with clinical parameters.

Frequencies of OX40 **(a)** and OX40L **(b)** positive (SFI) blasts according to the different FAB classifications (single values, median, Kruskal-Wallis test) are showed. The expression (SFI) of OX40 **(c)** and OX40L **(d)** was analyzed on FAB M0-M2 vs. FAB M4-M5 (min/max whiskers, Mann-Whitney-U test). The expression (SFI) of OX40 **(e)** and OX40L **(f)** was analyzed primary (pAML) vs. secondary (sAML) AML (min/max whiskers, Mann-Whitney-U test). Distribution of OX40 **(g)** and OX40L **(h)** expression (SFI) throughout NCCN risk group (min/max whiskers, Kruskal-Wallis test) is showed.

**Table S1**: therapies implemented for OX40^low^ and OX40^high^ patients

|  | **Number of patients (%)** |  | **Number of patients (%)** | **p-value** |
| --- | --- | --- | --- | --- |
| **OX40^low^** | 40 (100) | **OX40^high^** | 30 (100) |  |
| Age <65y | 20 (50) | Age <65y | 15 (50) | 1.000 |
| Age>65y | 20 (50) | Age>65y | 15 (50) | 1.000 |
| Induction therapy | 27 (68) | Induction therapy | 19 (63) | 0.801 |
| Allogenic transplantation | 16 (40) | Allogenic transplantation | 11 (37) | 0.808 |
| Palliative therapy* | 13 (32) | Palliative therapy* | 11 (37) | 0.801 |
|  |  |  |  |  |
| Palliative therapy* = demethylating substances or best supportive care | | | |  |

**Table S2**: therapies implemented for AML patients, stratified according to their positivity for OX40

|  | **Number of patients (%)** |
| --- | --- |
|  |  |
| **OX40 positive** |  |
| **1^st^ quartile** | **17 (100)** |
| Age <65y | 9 (53) |
| Age>65y | 8 (47) |
| Induction therapy | 11 (65) |
| Allogenic transplantation | 8 (47) |
| Palliative therapy* | 6 (35) |
|  |  |
| **2^nd^ quartile** | **17 (100)** |
| Age <65y | 7 (41) |
| Age>65y | 10 (59) |
| Induction therapy | 11 (65) |
| Allogenic transplantation | 5 (29) |
| Palliative therapy* | 6 (35) |
|  |  |
| **3^rd^ quartile** | **19 (100)** |
| Age <65y | 10 (53) |
| Age>65y | 9 (47) |
| Induction therapy | 12 (63) |
| Allogenic transplantation | 8 (42) |
| Palliative therapy* | 7 (37) |
|  |  |
| **4^th^ quartile** | **17 (100)** |
| Age <65y | 9 (53) |
| Age>65y | 8 (47) |
| Induction therapy | 12 (71) |
| Allogenic transplantation | 6 (35) |
| Palliative therapy | 5 (29) |
|  |  |
| Palliative therapy* = demethylating substances or best supportive care | |
